# A common trajectory of gut microbiome development during the first month in healthy neonates with limited inter-individual environmental variations

**DOI:** 10.1038/s41598-024-53949-6

**Published:** 2024-02-08

**Authors:** Xing-An Wang, Ju-Pi Li, Maw-Sheng Lee, Shun-Fa Yang, Yi-Sheng Chang, Ling Chen, Chang-Wei Li, Yu-Hua Chao

**Affiliations:** 1 Department of Pediatrics, Chung Shan Medical University Hospital, No. 110, Sec. 1, Chien-Kuo N. Road, Taichung, 402 Taiwan; 2 Institute of Medicine, Chung Shan Medical University, Taichung, Taiwan; 3 School of Medicine, Chung Shan Medical University, Taichung, Taiwan; 4 Department of Obstetrics and Gynecology, Lee Women’s Hospital, Taichung, Taiwan; 5 Department of Medical Research, Chung Shan Medical University Hospital, Taichung, Taiwan; 6 Department of Research and Development, AllBio Life Incorporation, Taichung, Taiwan; 7 Department of Clinical Pathology, Chung Shan Medical University Hospital, Taichung, Taiwan

**Keywords:** Developmental biology, Gastroenterology, Medical research

## Abstract

The early development of the gut microbiome is governed by multiple factors and has significantly long-term effects on later-in-life health. To minimize inter-individual variations in the environment, we determined developmental trajectories of the gut microbiome in 28 healthy neonates during their stay at a postpartum center. Stool samples were collected at three time points: the first-pass meconium within 24 h of life, and at 7 and 28 days of age. Illumina sequencing of the V3–V4 region of 16S rRNA was used to investigate microbiota profiles. We found that there was a distinct microbiota structure at each time point, with a significant shift during the first week. *Proteobacteria* was most abundant in the first-pass meconium; *Firmicutes* and *Actinobacteria* increased with age and were substituted as the major components. Except for a short-term influence of different delivery modes on the microbiota composition, early microbiome development was not remarkably affected by gravidity, maternal intrapartum antibiotic treatment, premature rupture of membranes, or postnatal phototherapy. Hence, our data showed a similar developmental trajectory of the gut microbiome during the first month in healthy neonates when limited in environmental variations. Environmental factors external to the host were crucial in the early microbiome development.

## Introduction

Ecological succession within the human gut is a dynamic process, from a nascent microbiome at, or perhaps even before, the time of birth to the ultimate equilibrium of an adult microbiome profile. The development of the gut microbiome during early life has significant and lasting effects on later-in-life host physiology and even on the occurrence of various diseases^1–5^. Therefore, research on its evolution during early childhood has recently received a lot of attention.

The infant gut is initially colonized by diverse microbes, mainly coming from the mother and the surrounding environment during the perinatal period^6–8^. Thereafter, the gut microbiome undergoes a maturation process with great shifts in both composition and diversity with increasing age. It has been proposed that the gut microbiome undergoes most of its development very early in life^9,10^. Therefore, it is important to know the reference founder from which the subsequent gut microbiome is derived. However, limited data are available regarding the microbial signatures of the first-pass meconium.

It has become increasingly clear that an individual’s gut microbiome appears to be shaped by multiple factors from the host and the environment and is modulated by age over time^11–13^. It is useful to minimize differences in the nursing environment among participants to examine the effects of perinatal characteristics on the temporal development of the gut microbiome in early life. Here, we aimed to determine developmental trajectories of the early gut microbiome in healthy neonates with minimal inter-individual variations in the environment. Taking advantage of the traditional Chinese ritual of postpartum confinement, all babies and their mothers in the present study stayed in a single care center during the entire study period and stool samples were collected at three different time points within 1 month after birth.

## Results

### Study cohort

A total of 28 neonates fulfilled all the inclusion criteria and had complete collection of stool samples during their stay at the postpartum care center. This study included 12 males and 16 females. Intrapartum antibiotic treatment was used in fifteen neonates for maternal Group-B Streptococcus positivity or premature rupture of membranes (PROM). PROM was noted in ten neonates, and the duration ranged from 30 min to 40 h. Based on the baby-friendly policy, all neonates received breast feeding initially. Additional infant formula was given if needed. The proportion of breast milk was tending upwards with age but cannot be determined accurately. The clinical data were outlined in Table 1.

**Table 1:** Descriptive data of the study population.

Variable	*n* = 28
Gender	
Male	12
Female	16
Gestational age (weeks)	38.3 (35.2–40.0)
Birth weight (g)	3010 (2020–3620)
Mode of delivery	
Vaginal	15
C-section	13
Primigravida	6 (21.4%)
Mother’s age (years)	33.9 (23.9–45.9)
Maternal intrapartum antibiotic treatment	15 (53.6%)
Premature rupture of membranes (PROM)	10 (35.7%)
Duration (hours)	6.5 (0.5–40.0)
Phototherapy for neonatal jaundice	6 (21.4%)
Feeding style	
Exclusive breast feeding or formula feeding	0
Mixed (breast + formula feeding)	28 (100%)

Data were shown as number (percentage) and median (range). Premature rupture of membranes is the rupture of gestational membranes prior to the onset of labor.

In Chinese culture, women rest for a month after they have a baby; this is termed postpartum confinement. Nowadays, a certain proportion of women in Taiwan do so at postpartum care centers, where adequate meals are provided for them and trained personnel help to take care of their babies^14,15^. The centers meet all the needs of new mothers and their babies, and they stay indoors all day during the period of postpartum confinement. Various environmental factors may affect the gut microbiome in humans. To minimize these differences, a cohort of neonates who were born in a single hospital and stayed in a single postpartum care center during a certain period was enrolled in the study. During they stayed at the care center, stool samples were collected at three time points: the first-pass meconium, and at 7 and 28 days of age. All the 28 neonates spontaneously evacuated the first-pass meconium after birth but not later than 24 h.

### A distinct microbiota community structure at each time point

Total DNA was extracted from the fecal samples, and the least DNA extraction yield among these samples was 169.2 ng. As known, there are a low number of bacteria within the first-pass meconium, but it is impossible to obtain meconium via a sterile procedure. In our previous study, samples collected from blank diapers were used for sequencing control to assess contamination. We found distinct microbiota community structures in the first-pass meconium samples and control samples^16^, implicating that the procedure of sample collection in our study was reliable.

**Figure 1:**
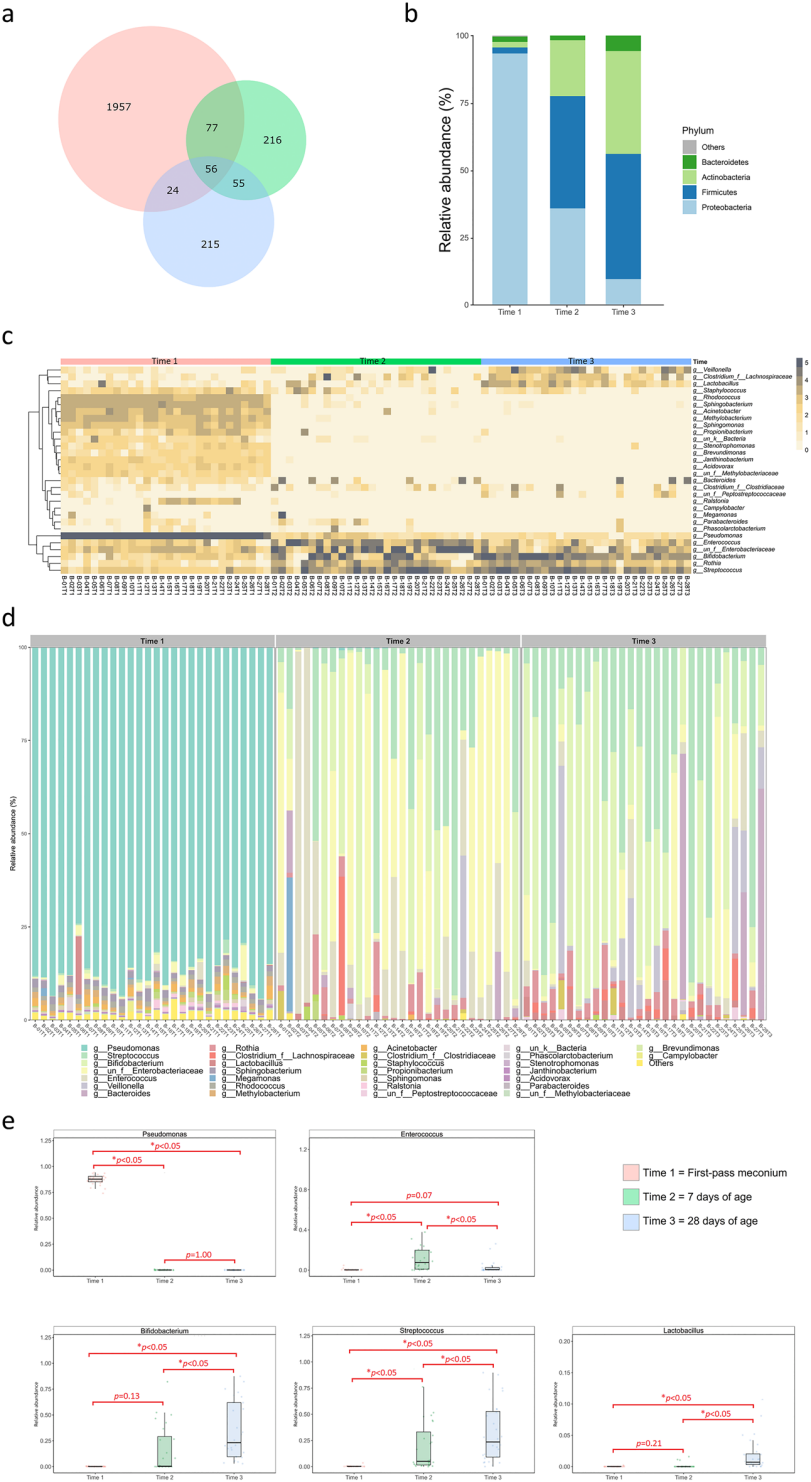
The evolution of the gut microbiota composition during the first month of life. (**a**) Venn diagram. (**b**) Mean relative abundances of the dominant taxa (at the phylum level) at each time point. (**c**) Heatmap of the top 30 OTUs at the genus level. (**d**) Bar plot showing the relative abundances of the annotated bacterial genera in each sample. The 30 most abundant genera across all samples were shown, with the remaining minor genera combined in the group “Others”. (**e**) Changes in the relative abundances of the five dominant genera over time. The box was drawn from the first quartile to the third quartile with a horizontal line in the middle to denote the median.

We sequenced and analyzed all 84 stool specimens from the 28 neonates. Sequencing generated an average of 174,425 reads per sample, and 2600 operational taxonomic units (OTUs) were identified. The Venn diagram displayed the number of OTUs that were shared or exclusive at different time points (Fig. 1a). Only 56 of the 2600 OTUs were shared across the three time points, suggesting the presence of a distinct microbiota structure at each time point. In particular, 92.6% of OTUs in the first-pass meconium within 24 h of life were unique. Figure 1b revealed the evolution of the gut microbiota composition at the phylum level. *Proteobacteria* was most abundant in the first-pass meconium, but the relative abundance decreased significantly at 7 and 28 days of age (*p* < 0.05). *Firmicutes* and *Actinobacteria* increased significantly with age (*p* < 0.05) and were substituted as the major components. In addition, the abundance of *Bacteroidetes* gradually increased with increasing age (*p* < 0.05).

As shown in Fig. 1c, taxonomic analysis identified the top 30 OTUs with the highest abundance at the genus level. A significant shift in the gut microbiota composition was noted during the first week of life. Among all meconium samples, *Pseudomonas* was the most abundant genus, accounting for > 80% of all sequences (Fig. 1d). Subsequently, the frequency of *Pseudomonas* declined significantly in stool samples collected at 7 and 28 days of age. Meanwhile, several enriched genera in the first-pass meconium, such as *Rhodococcus*, *Sphingobacterium*, *Acinetobacter*, *Methylobacterium*, and *Sphingomonas*, decreased abruptly in samples at 7 and 28 days of age. In contrast, *Enterococcus*, *Bifidobacterium*, *Streptococcus*, and *Lactobacillus* were found to be enriched after the first week of life (Fig. 1e).

**Figure 2:**
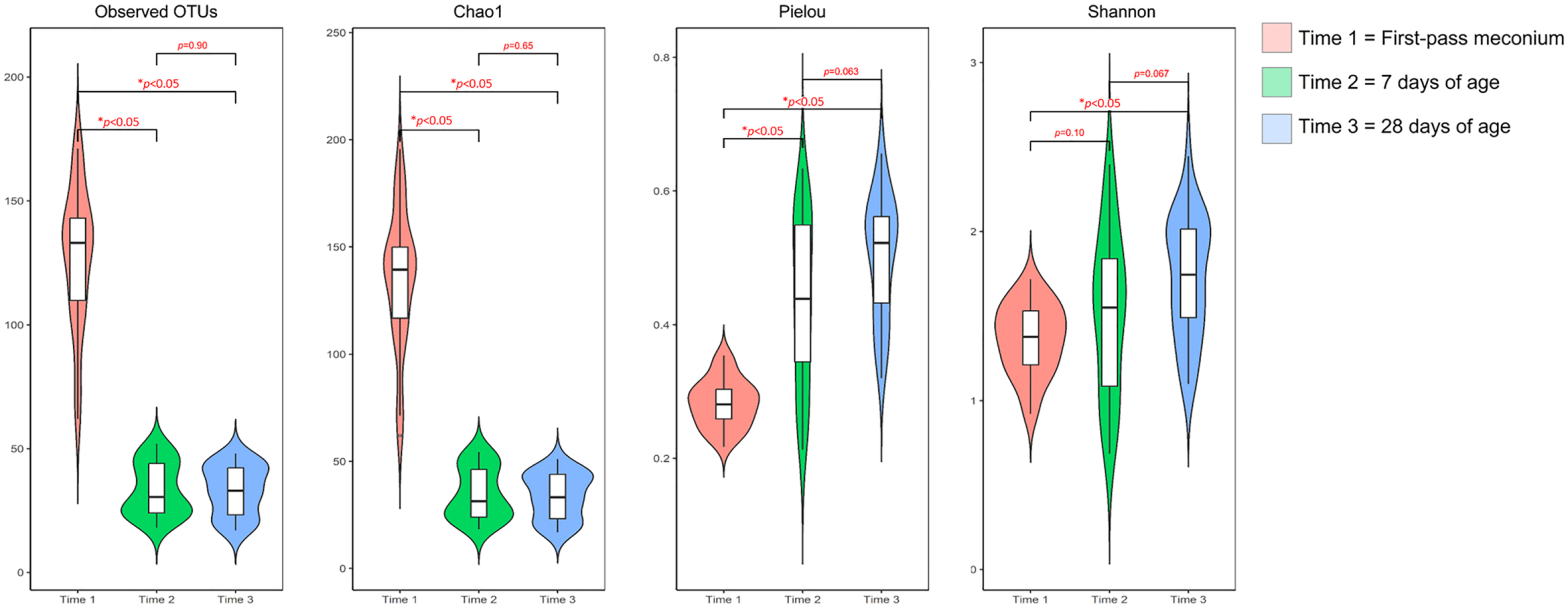
Alpha diversity indices at each time point showing the evolution of the microbial diversity in the gut ecosystem over time. The box was drawn from the first quartile to the third quartile with a horizontal line in the middle to denote the median. The whiskers indicated variability outside the upper and lower quartiles.

Alpha diversity was used to profile the microbial richness and evenness of the gut ecosystem (Fig. 2). Compared with samples collected at 7 and 28 days of age, the number of observed OTUs was higher in the first-pass meconium samples. Consistent with the higher Chao1 index, the species richness was greater in the meconium. However, the Pielou index of the first-pass meconium samples was significantly lower, suggesting less evenness in the microbial communities within the meconium. Considering both richness and evenness, the Shannon diversity index of stool samples showed an upward trend with increasing age, suggesting that maturation of the gut microbiome is associated with an increase in diversity.

**Figure 3:**
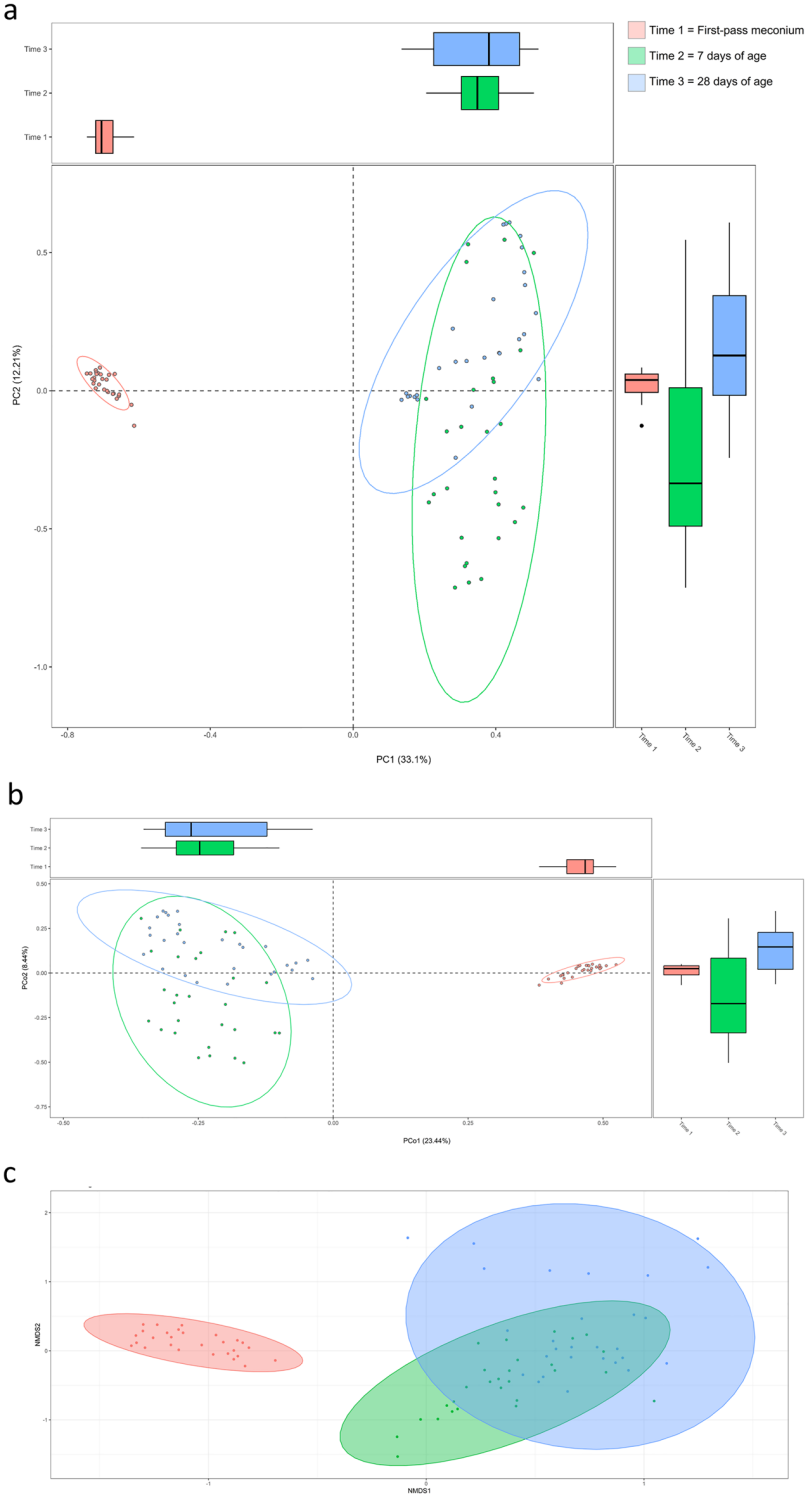
Beta diversity of the gut microbiome showing different microbial signatures at different time points. Each dot represented a sample. (**a**) Principal components analysis (PCA). (**b**) Principal coordinates analysis (PCoA). (**c**) Non-metric multidimensional scaling (NMDS).

Beta diversity was calculated to evaluate the differences in the microbiota community structure across different time points. As shown in Fig. 3a, principal components analysis (PCA) of predicted microbial functions revealed that the meconium samples were highly concentrated and distinct from the samples collected at 7 and 28 days of age. Illustrating the dissimilarity in overall composition by principal coordinates analysis (PCoA), the Bray–Curtis distance-based similarity analysis indicated that the largest changes in the microbiota composition occurred during the first week of life (Fig. 3b). The first-pass meconium samples were clustered separately from the samples of 7 and 28 days of age, and the Bray–Curtis distances between the samples of 7 and 28 days old were smaller. Concordantly, non-metric multidimensional scaling (NMDS) analysis revealed a significant difference in the distance between the first-pass meconium within 24 h of life and stool collected at 7 and 28 days of age (Fig. 3c).

**Figure 4:**
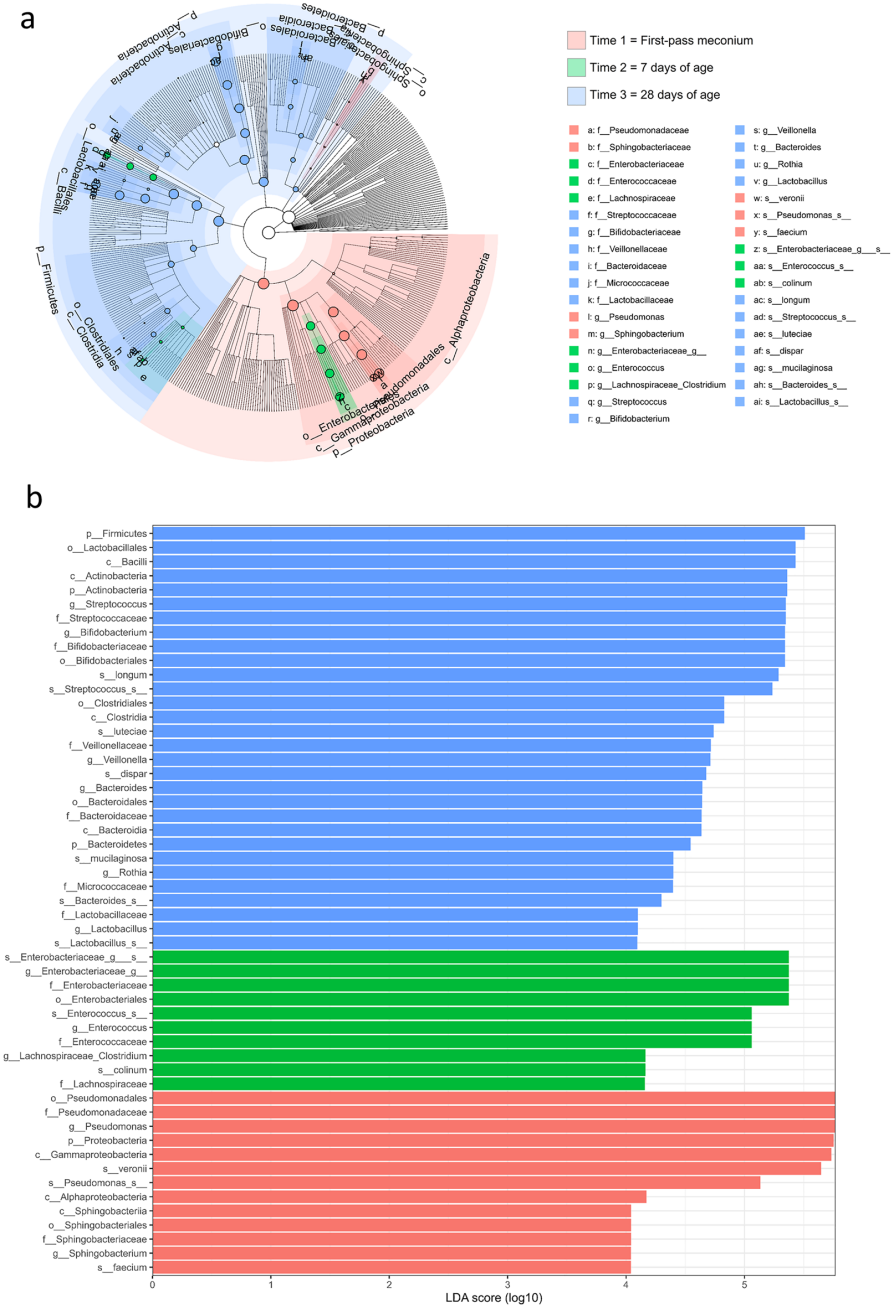
Linear discriminant analysis of effect size (LEfSe) to identify the most differentially abundant taxa at each time point. (**a**) Taxonomic cladogram. (**b**) Taxa meeting a significant linear discriminant analysis (LDA) threshold value of > 4.

As shown in Fig. 4, linear discriminant analysis of effect size (LEfSe) revealed that the dominant taxa across different time points were significantly different. A representative cladogram of the most differentially abundant taxa at each time point indicated shifts in the gut microbiota composition during the first month of life (Fig. 4a). At the genus level, *Pseudomonas* and *Sphingobacterium* were biomarkers for the first-pass meconium within 24 h of life, whereas *Bifidobacterium*, *Streptococcus*, *Veillonella*, *Bacteroids*, *Rothia*, and *Lactobacillus* were biomarkers at 28 days of age (Fig. 4b).

**Figure 5:**
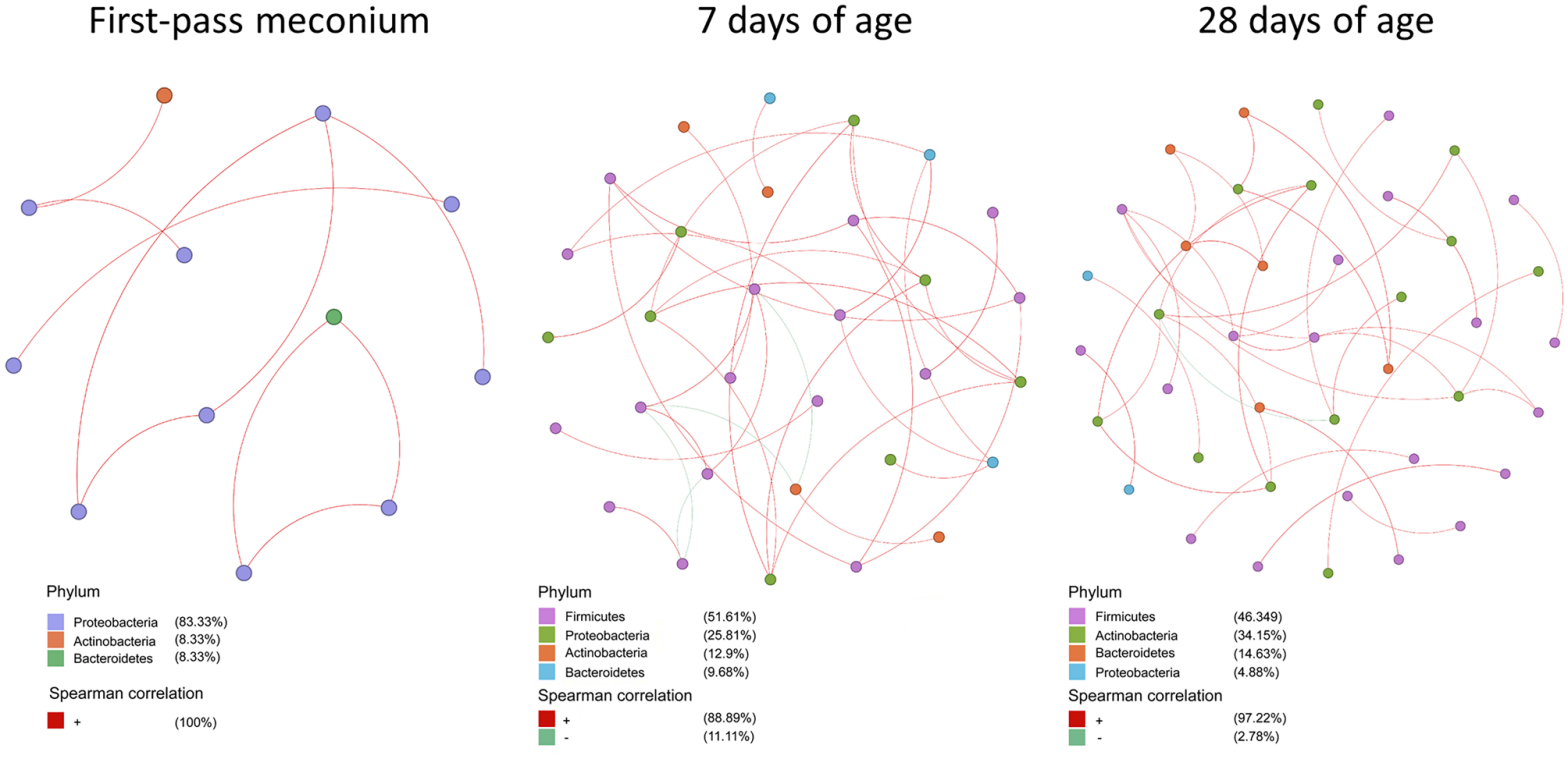
Co-occurrence network analysis constructed using 28 samples for each time point. Each node represented a taxon (at the phylum level); red and green edges between the nodes represented positive and negative correlations between taxa. Spearman coefficient correlations with a score lower than -0.6 and higher than 0.6 were considered significant.

The co-occurrence network illustrated how the evolution of the gut microbiome occurred during early life (Fig. 5). At the phylum level, the relative abundances of *Bacteroidetes* and *Firmicutes* increased with age, whereas *Proteobacteria* decreased significantly over time. Moreover, *Firmicutes* became the main fixed flora after seven days of age, with a large proportion of positive network associations.

### Effects of clinical factors on the development of the gut microbiome during the first month of life

**Figure 6:**
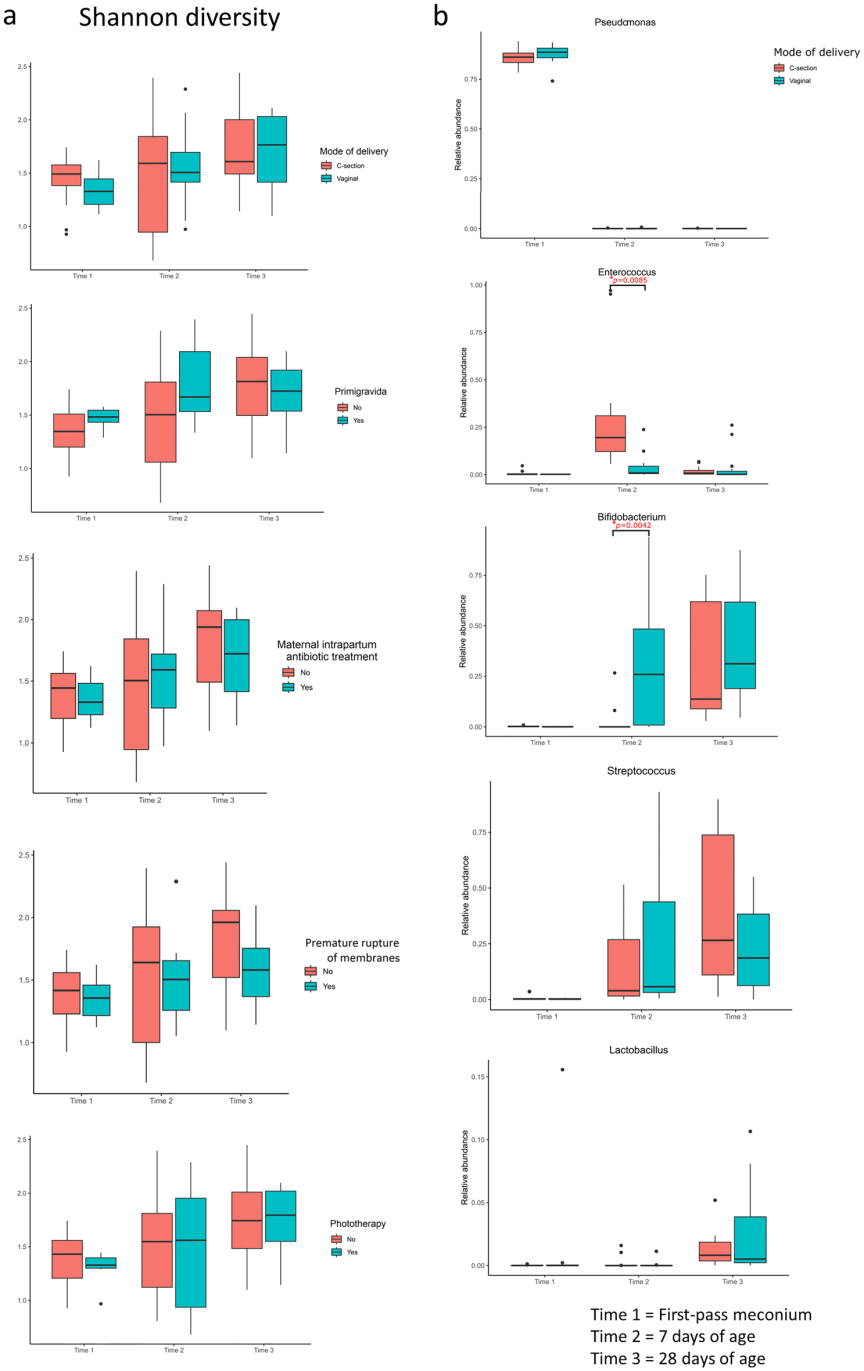
Relationship between clinical factors and the gut microbiome development. (**a**) Effects of multiple perinatal characteristics on alpha diversity at different time points. (**b**) Effects of different delivery modes on the relative abundances of the five dominant genera at different time points. The box was drawn from the first quartile to the third quartile with a horizontal line in the middle to denote the median. The whiskers indicated variability outside the upper and lower quartiles.

The influence of multiple perinatal characteristics on the development of the early gut microbiome was further evaluated. As shown in Fig. 6a, the Shannon diversity index exhibited an upward trend with age, but the increase was not affected by the mode of delivery, gravidity, maternal intrapartum antibiotic treatment, PROM, or postnatal phototherapy for neonatal jaundice. Next, effects of each clinical factor on the relative abundances of five dominant genera, including *Pseudomonas*, *Enterococcus*, *Bifidobacterium*, *Streptococcus*, and *Lactobacillus*, were determined. Compared with vaginally delivered infants, delivery by C-section was associated with a higher relative abundance of *Enterococcus* and lower relative abundance of *Bifidobacterium* at 7 days of age, but the association strength became insignificant at 28 days of age (Fig. 6b). Changes in the relative abundances of the five bacterial genera were not significantly affected by gravidity, maternal intrapartum antibiotic treatment, PROM, or postnatal phototherapy for neonatal jaundice (Supplementary Fig. 1). While there was limited sample size in our study, our data showed early microbiome development was not remarkably affected by these perinatal factors in the limited environment, except for a short-term influence of different delivery modes on the microbiota composition.

## Discussion

The human gut acquires a nascent microbiome around the time of birth and starts a transition to the ultimate equilibrium of an adult microbiome profile. While the development of healthy gut microbiota in early childhood has been somewhat characterized^17–19^, limited data are available on the longitudinal analysis of the gut microbiota structure from the time of birth. In this case cohort study, the gut bacterial composition was identified using 16S rRNA sequencing in stool samples from 28 healthy newborns collected at three time points: the first-pass meconium within 24 h of life, and at 7 and 28 days of age. Our results showed that transient colonization of non-pathogenic commensal microbiota from the *Proteobacteria* phylum was noted in the first-pass meconium. With increasing age, *Firmicutes* and *Actinobacteria* increased significantly and were substituted as the major components. Our study extends previous research with further characterization of the gut microbiome in very early life^20–22^. Due of the distinct microbiota community structure of the first-pass meconium, the establishment of the gut microbiome may not originate from the expansion of initial bacterial populations within the first-pass meconium.

A growing body of evidence suggests that the gut microbiome undergoes most of its development very early in life^9,10^. However, the evolution in healthy infants during very early life has not been fully elucidated. Unlike sampling only once during the first month of life in most studies, stool samples were collected at three time points within 1 month after birth in the present study. We observed that the overall composition of the gut microbiota changed markedly over time, with the most abrupt population change occurring during the first week of life. The analysis at the genus level showed that the alteration during the first week was mainly driven by *Enterococcus* and *Streptococcus* for the phylum *Firmicutes* and by *Bifidobacterium* for the phylum *Actinobacteria*. After the initial period, the relative abundances of *Bifidobacterium* and *Streptococcus* continued to increase, along with a remarkable increase in the abundances of *Veillonella*, *Bacteroids*, *Rothia*, and *Lactobacillus*. Meanwhile, we observed a gradual increase in Shannon diversity over time. It is understood that younger individuals have a lower diversity index compared to older children and adults^11^. Our result is consistent with previous reports^17–19^, and an increasing diversity is strongly associated with the development toward a mature microbiome.

The evolution of the gut microbiome after birth is governed by multiple host factors and factors external to the host^11–13^. For a study to investigate the influence of maternal and infant characteristics on the early microbiota development, it is especially important to minimize differences in the nursing environment among participants. However, it is very difficult to restrict the activities of mothers and their babies for study. Nearly all related studies in the literature have collected stool samples from babies by their parents at home. Certainly, there are wide inter-individual variations in the surrounding environment. As known, the intestinal microbiome in children under 3 years of age is highly dynamic^11^. In the early life, there was a pronounced level of inter-individual variation in bacterial species assemblages over time from baby to baby, even those apparently healthy^23^. The traditional Chinese ritual of postpartum confinement carried out in a single postpartum care center provided a special opportunity for us to uniform the most influential factors of the environment, including foods of the mothers and their babies^14,15^. From the delivery room to the baby room, all neonates in the present study received the same perinatal and postnatal care in a single building. All stool samples were collected by highly-trained nurses during their stay at the nursing center. Moreover, their mothers received the same postpartum care at the center and were kept indoors during the entire study period. The diet for the mothers and infant formula for the babies were chiefly provided by the center, and all babies in the present study received a similar feeding pattern. In contrast to the results of previous studies which showed great differences in the dynamics of the gut microbiome^11,23,24^, we found that these neonates shared a common developmental trajectory of the gut microbiome during the first month of life in the limited environment. Although the small sample size, our data showed that early microbiome development was not significantly affected by gravidity, maternal intrapartum antibiotic treatment, PROM, or postnatal phototherapy. Only a short-term influence of different delivery modes on the gut microbiota composition was observed. We speculate that the developmental trajectory of the early gut microbiome in healthy neonates may be governed principally by factors external to the host.

During the perinatal period, the infant gut is initially colonized by diverse microbes coming mainly from the mother and the external environment^6–8^. Our study showed that several environment-associated bacteria, such as *Pseudomonas* and *Sphingobacterium*, were first detected after birth, but soon a great shift in the gut microbiota composition occurred. Colonization by *Enterococcus*, *Bifidobacterium*, and *Streptococcus* was evident at 7 days of age, and the abundances of *Bifidobacterium*, *Streptococcus*, and *Lactobacillus* continued to increase with age. These bacteria may mainly originate from infant food, including breast milk and formula. As shown in the co-occurrence network (Fig. 5), the richness of these bacteria with increasing microbiota diversity is fundamental to the development of a healthy and mature gut microbiome, despite different development trajectories may go on^23,25^.

Some limitations are inherent in our study. First, the sample size of the cohort was small. Despite these infants shared a common developmental trajectory of the gut microbiome during the first month of life, our sample size may lack sufficient power to account for the influence of complex perinatal factors on early microbiome development. Second, our study did not enroll control infants who stayed at home after birth. Nevertheless, this observational study was designed to investigate early microbiome development in healthy infants with limited inter-individual environmental variations. Taking advantage of the unique Chinese tradition, our study was conducted in a postpartum care center. Finally, the impact of different feeding styles on the gut microbiome cannot be assessed. All babies in the present study received the same feeding pattern, and the proportion of breast milk cannot be determined.

In conclusion, we used a longitudinal birth cohort of 28 healthy infants during their stay at a postpartum care center to describe early microbiome development within 1 month after birth. Our study revealed a distinct microbiota composition at each time point. The greatest shift in the microbiota composition occurred during the first week. These infants shared a similar developmental trajectory of the gut microbiome during the first month of life in the limited environment.

## Materials and methods

### Study population and sampling

From December 2022 to January 2023, all pregnant women admitted to the Lee Women’s Hospital with the will for 28-day postpartum care at the Anxin Postpartum Nursing Home were invited to participate in this study at the time of admission to labor and delivery. Anxin Postpartum Nursing Home is affiliated with Lee Women’s Hospital and is responsible for the traditional Chinese ritual of postpartum confinement. This prospective case-cohort study was conducted in accordance with the Declaration of Helsinki and was approved by the Institutional Review Board of Chung Shan Medical University Hospital (CS2-22178). Written informed consent was obtained from all legal guardians of the participants.

Stool samples were collected by the child’s named nurse at three time points: the first-pass meconium, and at 7 and 28 days of age. Fresh stool samples were obtained from diapers after spontaneous evacuation and stored in a fecal collecting kit (AllBio Science, Taichung, Taiwan) at – 20 ℃. As soon as possible, the samples were transported to the laboratory at the AllBio Life Incorporation for extraction and sequencing. Clinical information relating to birth and postnatal care during the stay was also collected.

Newborn infants who fulfilled all the following criteria were eligible for data analysis: (1) babies were born to healthy mothers after normal pregnancy; (2) mothers did not receive probiotic supplementation during pregnancy; (3) the delivery process was uncomplicated and the Apgar scores were higher than 7; (4) babies were apparently normal without known congenital anomalies; (5) babies started oral feeds within the first 24 h; (6) babies did not require medical treatment except phototherapy; and (7) the first-pass meconium was spontaneously evacuated after birth.

### DNA extraction and sequencing

Total genome DNA from fecal samples was extracted using the EasyPure Stool Genomic DNA Kit (AllBio Science, Taichung, Taiwan), and DNA concentration was determined using the Qubit dsDNA HS Assay Kit (Thermo Fisher Scientific, Waltham, MA, USA). The samples were stored at – 20 °C for preservation before further PCR amplification and sequencing.

A sequencing library was constructed using the MetaVx Library Preparation Kit (Genewiz, South Plainfield, NJ, USA). The V3 and V4 hypervariable regions of prokaryotic 16S rDNA were selected to generate amplicons for taxonomic analysis. The forward primer sequence was ‘CCTACGGRRBGCASCAGKVRVGAAT’ and the reverse primers sequence was ‘GGACTACNVGGGTWTCTAATCC’. In the second-stage PCR, adapters and index sequences were added to either end of the amplified fragment. The library was purified using magnetic beads, and the concentration was determined using a microplate reader (Tecan Infinite 200 Pro). The fragment size was determined by agarose gel electrophoresis. Next-generation sequencing was conducted using the Illumina MiSeq Platform (Illumina, San Diego, CA, USA). Automated cluster generation and 250/300 paired-end sequencing with dual reads were performed, according to the manufacturer’s instructions.

### Bioinformatic analysis

The sequencing data were analyzed using QIIME2 (version 2021.11) with default parameters^26^. The DADA2 algorithm was used for quality filtering and merging sequences with greater than 97% similarity. Taxonomic assignment of each OTU was performed according to the Greengenes database, generating a table of amplicon sequence variants for further analysis. After taxonomic classification, random sampling was applied to flatten the number of sequences in all the samples. The taxa of the same type were agglomerated at the phylum, class, order, family, genus, and species levels.

Biodiversity was calculated at the OTU level using the R software and plotted using the MicrobiotaProcess package. Alpha diversity was used to investigate species richness and evenness within samples, as illustrated by the number of observed OTUs, Chao1, Pielou, and Shannon indices. For beta diversity, phylogeny-based UniFrac analysis was performed to evaluate the diversity and degree of difference among samples using the permutational multivariate analysis of variance (PERMANOVA) method. PCA was based on the taxonomic distribution; PCoA and NMDS were based on the Bray–Curtis distance matrix. LEfSe was applied to determine the taxa that best discriminated between the groups of samples. LEfSe was used for differential abundance analysis to generate cladograms and linear discriminant analysis (LDA) plots.

### Statistical analysis

Data analysis was performed using the R software. For continuous variables, the nonparametric Wilcoxon test was used to compare data of different time points. A value of *p* < 0.05 was considered statistically significant. Spearman coefficient correlations were used to exam co-occurrence network, and a score lower than -0.6 or higher than 0.6 was considered significant.

### Supplementary Information

Supplementary Figure 1.

## Data Availability

The datasets generated during the current study are available in the figshare repository, (https://figshare.com/articles/dataset/Dataset_underlying_the_research_Dynamics_of_early_gut_microbiome_during_the_first_month_of_life_in_healthy_neonates/23734455). Analyzed data were included in this article and its online supplementary material.
