# Supplementary material for: A common trajectory of gut microbiome development during the first month in healthy neonates with limited inter-individual environmental variations
Source: Sci Rep. 2024 Feb 8;14:3264. doi: 10.1038/s41598-024-53949-6 (PMC10853277; doi:10.1038/s41598-024-53949-6)
Supplement: Supplementary file 1 — Supplementary Figure 1. [file 41598_2024_53949_MOESM1_ESM.docx]

**
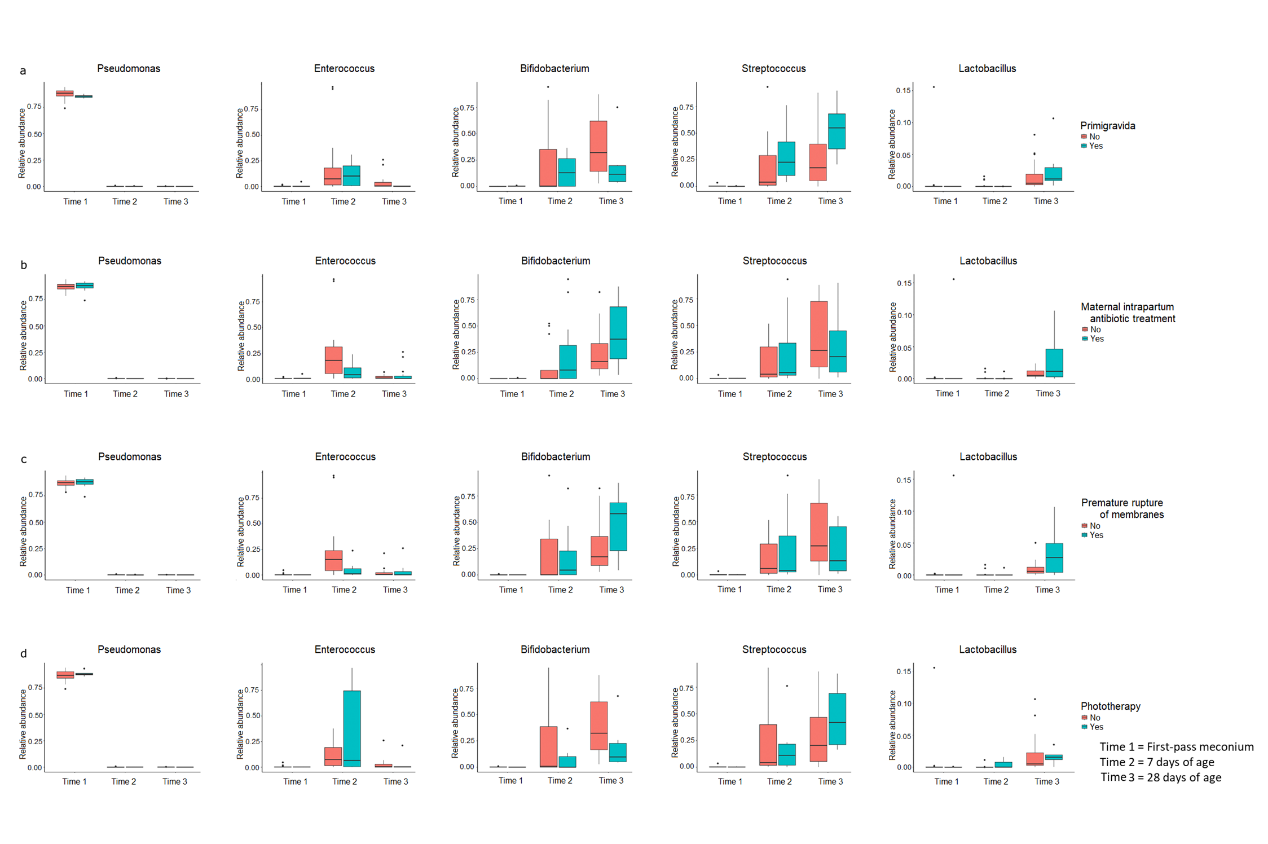
**

**Supplementary Figure 1.** Influence of each perinatal factor on the relative abundances of the five dominant genera over time. (a) Gravidity. (b) Maternal intrapartum antibiotic treatment. (c) Premature rupture of membranes. (d) Postnatal phototherapy for neonatal jaundice. The box was drawn from the first quartile to the third quartile with a horizontal line in the middle to denote the median. The whiskers indicated variability outside the upper and lower quartiles.
